# GPR35 acts a dual role and therapeutic target in inflammation

**DOI:** 10.3389/fimmu.2023.1254446

**Published:** 2023-11-16

**Authors:** Yetian Wu, Pei Zhang, Hongjie Fan, Caiying Zhang, Pengfei Yu, Xinmiao Liang, Yang Chen

**Affiliations:** ^1^ Ganjiang Chinese Medicine Innovation Center, Nanchang, China; ^2^ Division of Cancer Epidemiology and Genetics, National Cancer Institute, National Institutes of Health, Rockville, MD, United States; ^3^ CAS Key Laboratory of Separation Science for Analytical Chemistry, Dalian Institute of Chemical Physics, Chinese Academy of Sciences, Dalian, China

**Keywords:** GPR35, pro-inflammatory, anti-inflammatory, inflammatory diseases, therapeutic target

## Abstract

GPR35 is a G protein-coupled receptor with notable involvement in modulating inflammatory responses. Although the precise role of GPR35 in inflammation is not yet fully understood, studies have suggested that it may have both pro- and anti-inflammatory effects depending on the specific cellular environment. Some studies have shown that GPR35 activation can stimulate the production of pro-inflammatory cytokines and facilitate the movement of immune cells towards inflammatory tissues or infected areas. Conversely, other investigations have suggested that GPR35 may possess anti-inflammatory properties in the gastrointestinal tract, liver and certain other tissues by curbing the generation of inflammatory mediators and endorsing the differentiation of regulatory T cells. The intricate role of GPR35 in inflammation underscores the requirement for more in-depth research to thoroughly comprehend its functional mechanisms and its potential significance as a therapeutic target for inflammatory diseases. The purpose of this review is to concurrently investigate the pro-inflammatory and anti-inflammatory roles of GPR35, thus illuminating both facets of this complex issue.

## Introduction

Inflammation is a multifaceted physiological reaction that serves a critical role in the immune response against infection, tissue injury, and other provocations. It orchestrates a series of cellular and molecular activities with the ultimate goals of eradicating pathogens, repairing damaged tissues, and reinstating homeostasis (1). However, a dysregulated or persistent inflammatory response can pave the way for numerous diseases, encompassing autoimmune disorders where the body mistakenly attacks its cells, chronic inflammatory conditions that endure over extended periods, and even cancer where abnormal cells proliferate uncontrollably (2–4). The link between inflammation and such diverse diseases underscores the importance of understanding and effectively managing inflammation.

G protein-coupled receptors (GPCRs) constitute a vast family of cell surface receptors that fundamentally mediate cellular responses to a myriad of stimuli, including inflammatory (5, 6). Among these receptors, GPR35 has recently drawn considerable interest due to its suspected role in inflammatory processes. Discovered in 1998, GPR35 is highly expressed in the intestine and various immune cells. Owing to its wide expression and the significance of its function, it is perceived as a prospective therapeutic target for various diseases (7–9).

The involvement of GPR35 in inflammation is complex and context-dependent, demonstrating both pro-inflammatory and anti-inflammatory effects in disparate cellular and tissue settings. GPR35 has been implicated in modulating an array of inflammatory responses, including the activation of immune cells, production of cytokines, and chemotactic movements (10–12). It interacts with several signaling pathways, such as MAPK, NF-κB and others, thereby modulating inflammatory signaling dynamics. Furthermore, the functionality of GPR35 can be influenced by various ligands, ranging from endogenous metabolites to synthetic compounds, thus impacting its downstream effects in the inflammation process (13). Through these complex interactions and functions, GPR35 illustrates the multifactorial nature of inflammation and the intricate molecular dance that enables the body to respond appropriately to internal and external stressors. Yet, it also underscores the delicate balance that must be maintained, as dysregulated signaling via pathways involving GPR35 could potentially lead to pathological inflammation, underscoring the need for further research to elucidate its precise role and regulatory mechanisms.

In this comprehensive review, we explored the current understanding of GPR35, with a particular focus on its role in inflammation. We began by presenting a detailed overview of GPR35, discussing aspects such as its expression pattern, signaling mechanisms, and the variety of ligands it interacts with. From there, we investigated the pro-inflammatory role of GPR35, and its activation influences the immune response, including the impact on immune cell activation, cytokine production, and chemotactic activity within various immune cells such as neutrophils, macrophages, and invariant natural killer T (iNKT) cells. Subsequently, we discussed an in-depth examination of the anti-inflammatory role of GPR35 and covered aspects such as its impact on immune cell signaling, cytokine generation, and the resolution of inflammation. In addition, we explored the intricate signaling pathways and mechanisms underpinning the pro-inflammatory and anti-inflammatory effects that are triggered following GPR35 activation. This comprehensive exploration will serve as a map guiding us through the complicated roles of GPR35 in inflammation, shedding light on its significance and potential therapeutic value.

## Expression pattern of GPR35

GPR35 is classified as a class A orphan GPCR compromising nearly 85% of all GPCRs. GPCRs play a pivotal role in metabolite sensing within the intestine, acting as a significant connector linking the microbiota, immune system, and intestinal epithelium (14). Most GPCRs are predicted to be responsible for encoding olfactory receptors, while the rest are divided between receptors that bind with recognized endogenous compounds and those termed as orphan receptors because their specific endogenous activators remain unidentified (15).

Despite various endogenous ligands of GPR35, a definitive endogenous activator has not yet been firmly established, which maintains its status as an orphan receptor (13). Human GPR35 gene is transcribed and translated into three distinct variants (16). Both variants 2 and 3 encode the longer isoform GPR35b, which diverges from GPR35a by possessing an extended N-terminal sequence of 31 additional amino acids, thereby lengthening its extracellular domain. Despite this distinction, the subsequent sequences of the two isoforms are identical. mRNA expression of GPR35a and GPR35b have been identified in human tissues. Notably, GPR35b, which is highly expressed in gastric and colon cancer tissues, may have associations with carcinogenesis (17). Intriguingly, GPR35b demonstrates a lower agonist response efficacy than GPR35a (18). It was reported that the elongated N-terminus of the longer isoform may constrain G protein activation while boosting the interaction with β-arrestin (19).

GPR35 expression pattern suggests its pivotal role in immune responses and inflammation. GPR35 expression is particularly prominent in the small intestine and colon, with moderate but noticeable expression in the stomach, liver, spleen, kidney, and sympathetic neurons (20, 21). This widespread presence across various organ systems indicates its broad functionality within the body. Moreover, GPR35 expression is not limited to these organ systems. It also appears in various immune cells, including monocytes (CD14^+^), T-cells (CD3^+^), neutrophils, assorted dendritic cells, and invariant natural killer T cells (22–24), highlighting its integral role in immune responses. Among these, dendritic cells (CD103^+^/CD11b^−^) and macrophage clusters from the lamina propria and Peyer’s patch cells in mouse small intestine are noted for their high expression of GPR35 (25).

Furthermore, there is a significant upregulation of GPR35 in neutrophils and intestinal tissues during the invasion of pathogenic microbes, as well as in mast cells during the stimulation of IgE antibodies, indicating a pronounced inflammatory response (21, 26, 27). Additionally, GPR35 presence in epithelial and endothelial cells, which are key participants in the inflammatory response (28), further underscores broad impact across numerous cell types and physiological processes. This ubiquitous distribution and varied presence underscore GPR35 potential as a major player in immune responses and inflammation. GPR35 presents the intriguing features with tissue specificity and different isoforms, taking into consideration when examining the potential of GPR35 as a therapeutic target for inflammation-related conditions.

## GPR35 involved inflammation-related pathways

G proteins serve as the primary effector proteins for GPCRs. Typically, G proteins exist as a trimeric complex composed of α, β, and γ subunits. Upon activation of the GPCR, it binds to the Gα subunit and facilitates the exchange of GDP for GTP on Gα. This process leads to the disassociation of Gα from the Gβγ dimer. Following this separation, both Gα and Gβγ can independently initiate their respective signaling pathways (29).

**Figure 1:**
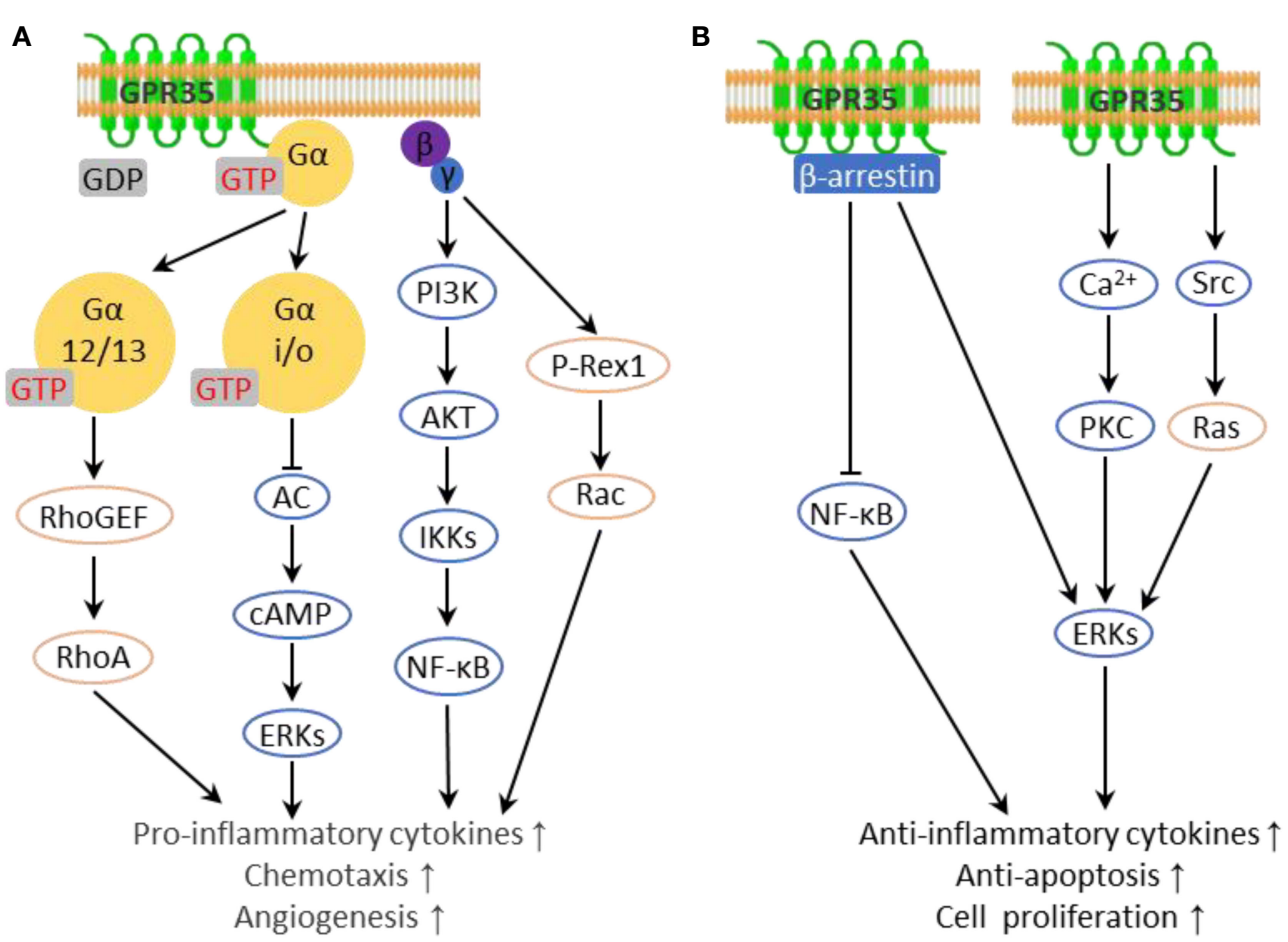
Inflammation-related downstream pathways of GPR35. **(A)** G proteins interacting with GPR35 transmit pro-inflammatory downstream pathways. **(B)** GPR35 transmits anti-inflammatory downstream pathways.

Four principal Gα protein families exist, and GPR35 has been found to couple predominantly with Gα12/13 and Gαi/o proteins (7, 24, 30) (**Figure 1A**). Gαi/o protein interacts with adenylate cyclase (AC), inhibiting its activity and consequently reducing intracellular cAMP levels, resulting in the dampening of the MAPK/ERK pathway (31). Interestingly, ERK may serve as an anti-inflammatory signal, suppressing the production of NF-κB dependent inflammatory factors through the inhibition of IKK activity (32). Gβγ dimer can activate phospholipase C beta (PLCβ), which results in phosphoinositide generation and activation of the PI3K/AKT pathway. This leads to the activation of a host of downstream transcription factors, notably NF-κB, a major player in the expression of genes encoding inflammatory factors (33). In the case of Gα12/13, activation leads to Rho-mediated cytoskeleton reorganization, collaborating with Gβγ to regulate immune cell chemotaxis to inflammation sites - these actions primarily result in pro-inflammatory effects (34, 35). Gαq coupled with GPR35 directly inhibits the PI3K catalytic subunit and then suppresses Akt activation. Additionally, Gαq plays a role in the activation of ERK through the PLCβ/Ca^2+^/Src signaling cascade (19, 36).

GPR35 has been demonstrated to directly engage with β-arrestin upon activation by agonists, leading to its internalization and desensitization (37). Beyond that, β-arrestins also function as signaling scaffolds, interacting with various pathways including the c-Jun N-terminal kinase, protein kinase B, and the extracellular signal-regulated kinase (ERK1/2) pathway in a G protein independent manner, leading to anti-inflammatory effects (38–40). In addition, β-arrestins interact with IκBα, leading to the suppression of NF-κB activation (41). It is worth noting that there are certain ligands capable of activating GPR35 without facilitating the interaction between GPR35 and β-arrestin, such as kynurenic acid (19, 42), 5-HIAA (42) and DHICA analogues (43). Moreover, GPR35 interacts with the sodium-potassium pump (Na/K-ATPase), a vital regulator of cellular electrochemical gradients and Src family kinase signaling (44). This interaction enhances Na/K-ATPase pump function, aiding in the regulation of Ca^2+^ homeostasis. Also, Na/K-ATPase directly activates the kinase Src, leading to ERK activation in macrophages and enterocytes (45). Given that elevated cytoplasmic Ca^2+^ levels promote ERK and NF-κB activation via PKC in inflammation, alterations in Ca^2+^ homeostasis have context-dependent impacts on inflammation (46, 47) (**Figure 1B**).

Furthermore, GPR35 has also been reported to modulate the production of reactive oxygen species (ROS), key mediators of inflammation. Under mechanical stress, GPR35 activation has been shown to enhance ROS production, and excessive ROS triggers further GPR35 expression (48). Conversely, GPR35 activation with Kynurenic acid in macrophages suppresses NLRP3 inflammasome activation and related inflammation by reducing mitochondrial damage and mitochondrial ROS production (49). These studies, despite showing divergent outcomes, reveal the significant role of GPR35 in modulating inflammation via ROS-mediated pathways.

In sum, a growing body of evidence proposes a sophisticated and intricate role of GPR35 in inflammation. It appears to exert both pro-inflammatory and anti-inflammatory influences, which depend on the type of cell, the specific signaling pathways, and the availability of ligands. The precise mechanisms through which GPR35 impacts inflammation-related pathways, however, remain somewhat elusive. Therefore, it necessitates additional research to comprehensively understand the subtleties of its signaling.

## Primary ligands with activity at GPR35

A pivotal element influencing the activity of GPR35 are its ligands, otherwise referred to as agonists and antagonists. Agonists are molecules that initiate signaling by binding to the receptor, thereby triggering downstream cellular responses. Conversely, antagonists exert opposing effects. Furthermore, the activation of receptors is not always fully “on or off” scenario while some agonists can activate the corresponding receptors in a biased manner. For instance, in the case of the kynurenic acid/GPR35 axis, while kynurenic acid triggers the activation of G proteins downstream of GPR35, it scarcely facilitates the interaction between GPR35 and β-arrestin (42, 43).

There are multiple single nucleotide polymorphisms (SNPs) located within the GPR35 gene linked with various immune and inflammation-related diseases, such as inflammatory bowel diseases, ankylosing spondylitis, and primary sclerosing cholangitis (9). One of the most prevalent variants, rs3749171, induces the replacement of a threonine with methionine in transmembrane domain III (T108M), demonstrating a significant correlation with inflammatory bowel disease (50). Although most SNP-induced variations do not significantly affect the potency of agonist ligands, the V76M variant (rs13387859) does exhibit a reduction in agonist potency (51). While this variant is present at a 2% allele frequency, it has not been associated with any disease.

In recent years, a multitude of both endogenous and synthetic GPR35 ligands have been discovered, enriching our understanding of this involvement in inflammation and a host of other biological phenomena (**Table 1**).

**Table 1:** A schematic representation of GPR35 ligands.

Name	Type	Potency (EC50)	Role in inflammation	References
Kynurenic acid	Metabolite of L-tryptophan	Human: 217 μM Rat: 66 μM	Anti-inflammatory	(52, 53)
LPA	Phospholipid derivative	Not reported	Pro-inflammatory	(54, 55)
5-HIAA	Serotonin metabolite	Not reported	Pro-inflammatory in Neutrophils	(26)
CXCL17	Endogenous peptide	Not reported	Anti-inflammatory	(56, 57)
cGMP	Cyclic nucleotide derived from GTP	Human: 131 μM	No conclusion	(58)
DHICA	Intermediate in the biosynthesis of elanin	Human: 22 μM	No conclusion	(40)
Reverse T3	Hormone produced in the thyroid gland	Human: 100 μM	No conclusion	(40)
Zaprinast	Synthetic agonist	Human: 2-8 μM Mouse: 1 μM Rat: 100 nM	No conclusion	(59)
Pamoic acid	Synthetic agonist	Human: 30-50 nM Mouse: Inactive Rat: >100 μM	Anti-inflammatory	(60, 61)
YE120	Synthetic agonist	Human: 30-35 nM	Anti-inflammatory	(62, 63)
Lodoxamide	Synthetic agonist	Human: 4 nM Rat: 13 nM	Allergic inhibition	(51)
Bufrolin	Synthetic agonist	Human: 13 nM Rat: 10 nM	Allergic inhibition	(51)
Compound 1	Synthetic agonist	Human: 26 nM Mouse: 17 μM Rat: 8 μM	No conclusion	(64)
PSB-13253	Synthetic agonist	Human: 12 nM Mouse: Inactive Rat: 1.4 μM	No conclusion	(65)
Amoxanox	Synthetic agonist	Human: 4 μM Rat: 23 nM	Mast cell stabilizer,anti-asthma and anti-allergy medication	(51)
Cromolyn disodium	Synthetic agonist	Not reported	Mast cell stabilizer, mitigate asthma	(21)
Nedocromil	Synthetic agonist	Human: 0.13 μM Mouse: 7.3 μM Rat: 2.7 μM	Mast cell stabilizer, mitigate asthma	(21)
Doxantrazole	Synthetic agonist	Human: 3.4 μM Rat: 300 nM	Mast cell stabilizer	(51)
Pemirolast	Synthetic agonist	Human: Inactive Rat: 95 nM	Mast cell stabilizer	(51)
Furosemide	Synthetic agonist	Human: 8.3 μM	Anti-inflammatory	(66)
Tyrphostin-51	Synthetic agonist	Human: 8 μM (DMR 120 nM)	No conclusion	(67)
2,3,5-THB	Synthetic agonist	Human: 8.4 μM (DMR 250 nM)	No conclusion	(68)
Gallic acid	Synthetic agonist	Human: 11.4 μM (DMR 1.16 μM)	Anti-inflammatory	(69)
wedelolactone	Synthetic agonist	Human: 1.39 μM (DMR 2.73 μM)	Anti-inflammatory	(69)
Ellagic acid	Synthetic agonist	Human: 2.96 μM (DMR 110 nM)	Anti-inflammatory	(69)
Aminosalicylate	Synthetic agonist	Not reported	Anti-inflammatory	(70)
Compound 50	Synthetic agonist	Human: 5.8 nM	No conclusion	(71)
CID2745687	Synthetic antagonist	Human (Ki): 10-20 nM	No conclusion	(60)
ML-145	Synthetic antagonist	Human (Ki): 25 nM	No conclusion	(72)

### Endogenous agonists

Kynurenic acid, a metabolite derived from tryptophan, is noted for its roles in the central nervous system, as well as anti-inflammatory property (73, 74). Its ability to activate GPR35 is significantly more potent in mice and rats compared to humans, where it requires 40- to 100-fold higher concentrations (20), occasionally failing to activate human GPR35 at high doses (27, 54). This species-selective aspect of the kynurenic acid/GPR35 axis has stirred discussion on whether this interaction is truly physiological, particularly in humans, leaving kynurenic acid as a potential endogenous ligand for GPR35 (7). Recently, it was demonstrated that pre-treatment with kynurenic acid mitigated the injuries sustained by both human iPS-cardiomyocytes and mouse cardiomyocytes following simulated ischemia/reperfusion (I/R) *ex vivo* (8). In mice, kynurenic acid stimulates the migration of CX3CR1^+^/GPR35^+^ macrophages in the small intestine, but not GPR35^-^ macrophages (75).

Lysophosphatidic acid (LPA), an active phospholipid derivative, is present in cell membranes and can be produced extracellularly to activate six known GPCRs including LPAR1-6 (76). It was displayed that the Ca^2+^ response triggered by 2-acyl LPA was significantly stronger in GPR35-expressed HEK293 cells compared to the response in the control cells, which pronounced difference was not observed when 1-acyl LPA was applied (54). GPR35 deficiency was found to inhibit LPA-induced Ca^2+^ signaling in bone marrow-derived macrophages (BMDMs). However, it was posited that GPR35 deficiency might compromise LPA signaling via other LPA receptors (45). Recently, GPR35 was affirmed as a potential LPA receptor linked to an inhibitory G protein (Gi) (27). However, there LPA failed to activate GPR35 in other experimental settings, which leaves the question open on whether LPA acts as an endogenous agonist for GPR35 (26, 77).

CXCL17, a homeostatic chemokine in mucosa, attracts dendritic cells and macrophages but can be expressed elsewhere during inflammation (78). It was reported that CXCL17 influenced GPR35 at nanomolar levels within a physiological range, unlike kynurenic acid (56). However, subsequent studies failed to show that CXCL17 induced migration or signaling responses in GPR35-expressing cells (79, 80). Further, the actions of CXCL17 in a neuropathic pain model in mice were decreased by kynurenic acid and zaprinast, suggesting the presence of a CXCL17 receptor other than GPR35 (81).

Recent findings indicate that 5-Hydroxyindoleacetic acid (5-HIAA), a metabolite of 5-hydroxytryptamine generated by activated platelets and extravascular mast cells, can act as an agonist for GPR35, promoting the recruitment of neutrophils (26) and the migration of eosinophils (82). Interestingly, this perspective offers fresh insight into the role GPR35 regulating pain sensations due to the association of 5-HIAA with pain and sensory neurons (83). Additionally, GPR35 plays a role in modulating cAMP production and inhibiting N-type Ca^2+^ channels in neurons and astrocytes, showcasing its potential involvement in pain management treatments (84). Moreover, other endogenous molecules such as 5,6-dihydroxyindole-2-carboxylic acid (DHICA), reverse T3 (3,3,5- triiodothyronine), and cyclic guanosine 3′-5′ monophosphate (cGMP) have demonstrated a degree of activity towards GPR35, albeit with modest potency. However, these observations require further validation (40, 58).

### Synthetic agonists

Zaprinast (2-(2-propyloxyphenyl) -8-azapurin-6-one), originally identified as a cGMP phosphodiesterase inhibitor, is one of the earliest discovered GPR35 ligands. Notably, the effects of Zaprinast on GPR35 can be separated from its cGMP phosphodiesterase inhibition properties with the intracellular calcium mobilization (59). With its moderate-to-high potency across human, mouse, and rat orthologs, Zaprinast has established its status as a go-to reference agonist for GPR35 research (30, 72). Its efficacy and wide applicability across different species have aided in comparative studies and offered insights into the roles GPR35 plays in a myriad of biological processes.

Pamoic acid (5-nitro-2-(3-phenylproplyamino) benzoic acid) emerged as a potential GPR35 ligand following screening exercises within the Prestwick Chemical Library. However, its activity at rat and mouse orthologs of GPR35 is noticeably less potent compared to its interaction with the human version. This lower efficacy significantly impedes its usage in these preclinical studies (30, 60). Despite this limitation, the discovery of pamoic acid interaction with GPR35 has contributed to the expanding catalog of ligands and may potentially prompt further development of more effective analogs, thereby expanding our understanding of GPR35 physiological role.

YE120 (2-(3-cyano-5-(3,4-dichlorophenyl)-4,5-dimethylfuran-2(5H)-ylidene) malononitrile) is another compound that was identified as a GPR35 agonist through dynamic mass redistribution (DMR) assays performed in the native cell line HT-29. Remarkably, it has demonstrated superior potency compared to zaprinast (62). This discovery indicates the continuing progress in our understanding of GPR35, providing a new promising candidate for the functionality and potentially enabling the development of more effective therapeutic strategies targeting GPR35.

Lodoxamide, a widely used anti-inflammatory mast cell stabilizer, is another synthetic agonist that targets GPR35. Its application in the treatment of allergic keratoconjunctivitis attests to its significant role in modulating inflammatory responses. However, despite its high potency towards human and rat GPR35, the effectiveness of lodoxamide is notably diminished to for the mouse orthologue, with its potency being a 100-fold lower (51, 85). This highlights the importance of species-specific investigations in understanding the precise role of GPR35 and its ligands in mediating inflammatory responses. Recently, there was a significant development in understanding the structural attributes of GPR35 through its structure when bound to a GPR35 agonist lodoxamide, which shows a novel site for divalent cation coordination and a distinctive ionic regulatory mechanism. This helps understanding the affinity of GPR35 for other anti-asthma and anti-allergy agents, especially those featuring symmetric diacid structures like lodoxamide. providing a clear pathway for the binding process (86)

Aminosalicylates, a first-line treatment for inflammatory bowel diseases (IBDs), have shown activity on both human and mouse GPR35, although their exact target remains undefined. Of these, the pro-drug olsalazine exhibits the greatest potency in terms of GPR35 agonism, promoting ERK phosphorylation and the translocation of β-arrestin2. Notably, in a model of dextran sodium sulfate (DSS)-induced colitis, the protective effects of olsalazine on disease progression and its inhibitory effect on TNFα mRNA expression, as well as the NF-κB and JAK-STAT3 pathways, are significantly reduced in mice with GPR35 knockout, thus suggesting a critical role of GPR35 in these anti-inflammatory actions (70).

Recently, a group of 2H-chromen-2-one derivatives has been identified as agonists for GPR35 using dynamic mass redistribution assays in HT-29 cells. The compound 6-Bromo-7-hydroxy-8-nitro-3-(1H-tetrazol-5-yl)-2H-chromen-2-one (Compound 50) emerged as the most potent GPR35 agonist with an EC50 of 5.8 nM (71). In another development, GPR35 fluorescent probes were designed based on known GPR35 agonists. These serve as valuable tools for GPR35 research and for the discovery of new synthetic GPR35 agonists. The most promising compound from this series exhibited the highest binding potency, along with a minimal nonspecific Bioluminescence Resonance Energy Transfer (BRET) binding signal, with a Kd value of 3.9 nM (87).

### Antagonists

CID2745687, known as methyl-5- [(tert-butylcarbamothioylhydrazinylidene)methyl] -1-(2,4- difluorophenyl)pyrazole-4-carboxylate, is a well-known antagonist of GPR35. It demonstrates the ability to obstruct the effects of the agonists pamoic acid and zaprinast in cells expressing either variant of human GPR35a or GPR35b. It is estimated that the inhibitor constant Ki is in the range of 10-20 nM, suggesting potential competitive function in a competitive manner (60). CID2745687 has a higher affinity for human GPR35 compared to its mouse and rat counterparts, limiting its application primarily to *in vitro* studies rather than rodent models (88). Another antagonist of GPR35 is ML-145, or 2-hydroxy-4- [4-(5Z)-5-[(E)-2-methyl-3- phenylprop-2-enylidene] -4-oxo-2-sulfanylidene-1,3- thiazolidin-3-yl] butanoylamino] benzoic acid. This compound also demonstrates high affinity for human GPR35 but shows appreciable affinity for the mouse and rat orthologues (72).

These antagonists and their specificity for the human GPR35 over rodent versions provide valuable insights into the diverse functionality and pharmacology of GPR35. They underline the challenges of studying GPR35 across different species but also offer opportunities for the development of novel therapeutic agents targeting the human GPR35 receptor.

## Pro-inflammatory roles of GPR35

**Figure 2:**
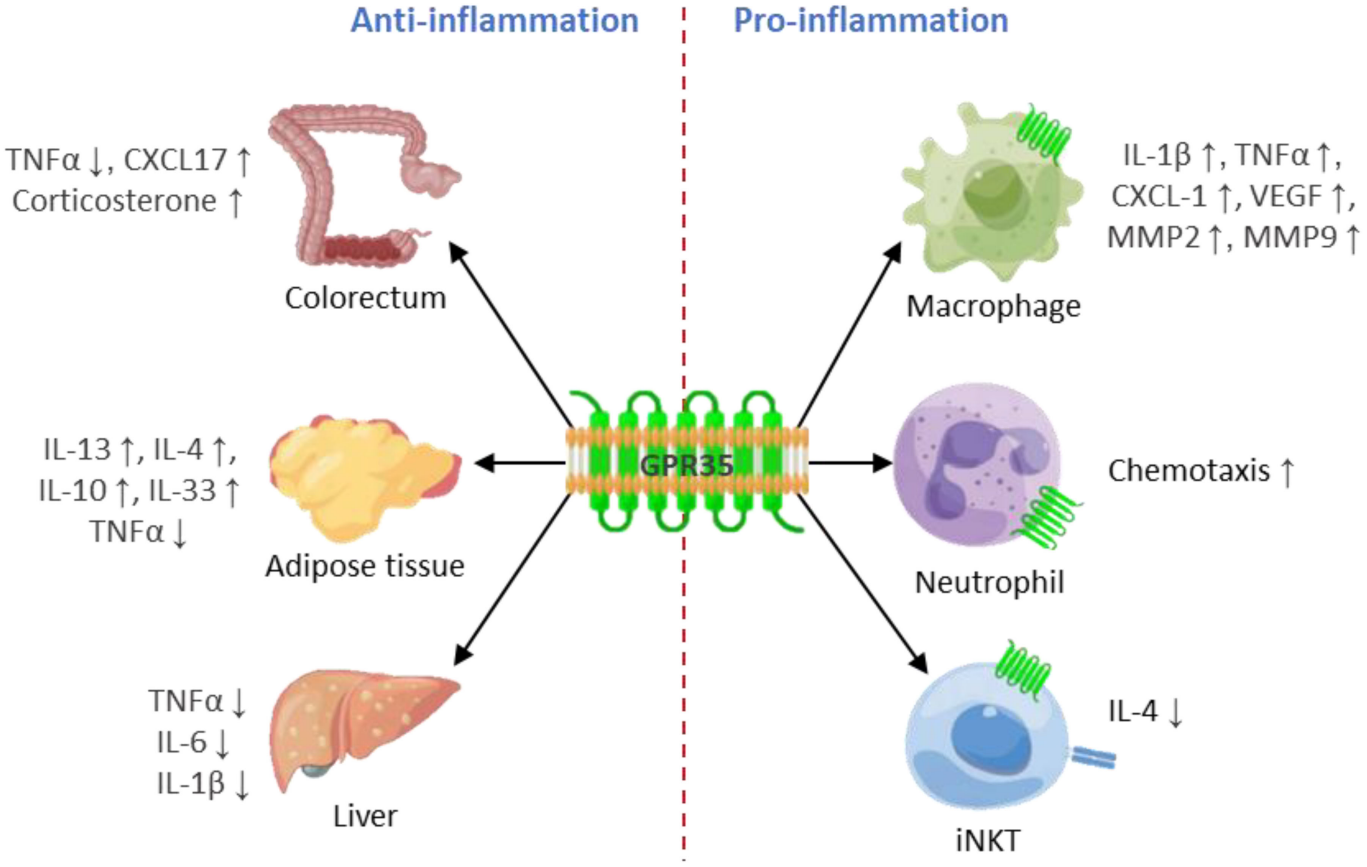
GPR35 plays a double-edged sword role in inflammation. GPR35 plays an anti-inflammatory role in colorectum, adipose tissue and liver, while plays a pro-inflammatory role in macrophages, neutrophils and iNKT.

GPR35 activation has been demonstrated to induce a pro-inflammatory state within cells, leading to increased production of pro-inflammatory cytokines and chemokines, which act as signal carriers, promoting the recruitment and activation of specific types of immune cells to the inflammation site (**Figure 2**). This process, in essence, highlights a crucial role of GPR35 in orchestrating immune responses, contributing to the exacerbation of inflammation, and potentially influencing the progression of various inflammatory conditions and diseases. There have been two diseases that are connected to the pro-inflammatory effects of GPR35. GPR35 plays a vital role in detecting bacteroides fragilis toxin and triggering an immune response in bacteroides fragilis toxin-induced colitis (89), and GPR35 loss in nucleus pulposus cells significantly reduces intervertebral disc degeneration typically triggered by inflammation induced by ROS or mechanical stress (49). In addition, encephalomyelitis (EAE) is worsened through the accumulation of GPR35^+^/Ly6C^+^ macrophages in the small intestine, while the blockage of KYNA-GPR35 signaling can alleviate EAE (75).

### GPR35 plays a pro-inflammatory role in neutrophils

Neutrophils, the most populous immune cells in human blood, act as first responders in the face of tissue damage and pathogenic infections. The journey of neutrophils from blood vessels to target tissues is complex, traditionally classified into four stages: 1. The egress of neutrophils from bone marrow into the bloodstream. 2. A multi-step adhesion cascade resulting in the congregation of adherent neutrophils in microvessels. 3. Diapedesis of neutrophils across the vascular wall, granting access to the extravascular space. 4. Interstitial migration of neutrophils to their ultimate target sites (90). Various small molecules and cytokines orchestrate these stages, resulting in a highly redundant regulatory system, which complicates the task of identifying dominant chemokines (91).

Interestingly, GPR35 expression is observed to be relatively low in inactive neutrophils, while it increases substantially in activated neutrophils, which aligns with the role of GPR35 in promoting neutrophil recruitment to inflammatory tissues (26). While GPR35 deficiency was found to somewhat dampen neutrophil adherence to blood vessel walls, this effect was not significantly observed in animal studies. Nevertheless, the role of GPR35 in neutrophil diapedesis has been identified as non-redundant (92). Moreover, 5-HIAA produced by activated platelets and extravascular mast cells, can activate GPR35 as an agonist, thus encouraging the recruitment of neutrophils (93).

In summary, through the complex interactions of these various mechanisms, GPR35 plays a significant role in promoting an inflammatory response in neutrophils by facilitating neutrophil recruitment, reinforcing its central role in the body response to inflammation.

### GPR35 plays a pro-inflammatory role in macrophages

Macrophages are specialized cells derived from monocytes in the bloodstream, and they play crucial roles in identifying, engulfing, and destroying bacteria, thereby initiating inflammation. These activated macrophages are classified into two types based on their involvement in inflammation: pro-inflammatory (M1) and anti-inflammatory (M2). M1 macrophages secrete pro-inflammatory cytokines such as IL-1, IL-6, TNF-α, as well as metalloproteinases MMP-2 and MMP-9, which help to reshape the extracellular matrix at inflammation sites. However, M2 macrophages produce IL-10, PDGF, IGF-1, TGF-β, and other cytokines that suppress inflammation and foster tissue repair (94–96).

*In vitro* experiments reveal that the absence of GPR35 does not influence the differentiation of macrophages into M1 and M2 subtypes. However, the level of secreted substances such as CXCL-1, VEGF, MMP2, and MMP9 is reduced in GPR35-deficient macrophages derived from mice with GPR35 deletion, specifically the LysM^+^ cells (44, 45). When observing macrophages in the colon, a notable difference in cytokine expression was seen between GPR35^+/+^ and GPR35^-/-^ macrophages. Proinflammatory cytokines like IL-1 and TNF were highly expressed in GPR35^+/+^ macrophages. Moreover, GPR35 was predominantly expressed in monocyte subgroups exhibiting high to medium levels of Ly6C in the intestine, with the level of intestinal TNF significantly dropping in GPR35^-/-^ macrophages (27). Ly6C^+^ macrophages, differentiated from monocytes expressing high to medium levels of Ly6C, are known to be pro-inflammatory (97).

Additionally, GPR35 deficiency hinders the adhesion of human peripheral monocytes induced by kynurenic acid (20). In contrast, the activation of GPR35 elevates the infiltration level of macrophages in gastric tissues (11). Taken together, it becomes evident that GPR35 contributes to the pro-inflammatory phenotype of macrophages.

### GPR35 has pro-inflammatory function in iNKT cells

Human iNKT cells constitute a unique subset of T cells that feature an invariant alpha-beta T-cell receptor (TCR) along with numerous surface molecules characteristic of natural killer (NK) cells. These iNKT cells can become activated either directly through the engagement of the invariant TCR with glycolipid antigens and CD1d, or indirectly through activated antigen-presenting cell (98). Notably, iNKT cells are critical immunoregulatory components capable of rapid, abundant cytokine production, thus influencing the behavior of other immune cells (99). iNKT cells have been found to express GPR35 at high levels. Following receptor activation, GPR35 undergoes internalization within these iNKT cells. It has been observed that specific GPR35 agonists significantly decrease the release of Interleukin-4 (IL-4), but not Interferon gamma (IFN-γ) (22). IL-4 secretion by iNKT cells plays a crucial role in anti-inflammatory processes, such as M2 polarization of macrophages, the transition of monocytes (from Ly6C^hi^ to Ly6C^lo^), and tissue repair, particularly in the liver (100–102). Thus, the activation of GPR35 in iNKT cells, which in turn reduces the release of IL-4, has the potential to stimulate inflammation.

Overall, it is increasingly apparent that GPR35 can play a pro-inflammatory role under certain circumstances. This is evidenced by the role of GPR35 in the stimulation of pro-inflammatory cytokines and chemokines production, the activation of inflammatory signaling pathways, and the amplification of pro-inflammatory responses in particular cell types. However, it is crucial to emphasize that the pro-inflammatory effects of GPR35 may be modulated by various factors. These include the microenvironment in which the receptor operates, the existence of other receptor types and signaling pathways, as well as the specific cellular type expressing GPR35. Further in-depth research is required to unravel the underlying mechanisms and conclusively establish the exact role of GPR35 within the context of inflammation. The complexity of these dynamics underscores the intricate interplay of processes within our immune responses and the need for a comprehensive understanding of GPR35.

## Anti-inflammatory role of GPR35

Although GPR35 is predominantly linked with pro-inflammatory actions across a range of immune cells, recent studies reveal its potential anti-inflammatory capabilities under certain circumstances. This perspective brings to light the anti-inflammatory roles of GPR35 (**Figure 2**), enriching our understanding of its part in maintaining immune homeostasis and potentially revealing novel therapeutic targets for treating various inflammatory conditions.

GPR35 displays an anti-inflammatory role, particularly in colonic inflammation. It is prominently expressed in the gastrointestinal tract, playing a pivotal role in regulating the healing process of gastrointestinal injuries. This means GPR35 can inhibit inflammation by maintaining gastrointestinal homeostasis. In fact, GPR35 demonstrates protective qualities in scenarios such as DSS-induced colitis (27, 63, 103). GPR35 agonists such as YE120, zaprinast, and pamoic acid have been shown to expedite wound repair in mouse colon epithelial cells by boosting cell migration through the augmentation of fibronectin expression and ERK phosphorylation (63). Interestingly, the recruitment of β-arrestin 2 and the activation of ERK1/2, mediated by pamoic acid, can be obstructed by the GPR35 antagonist CID2745687. The endogenous ligand of GPR35, 5-HIAA, has been recognized to mitigate the symptoms of ulcerative colitis, while the precise role of GPR35 was not delineated further (104). This insight suggests a potential therapeutic strategy, hinting that manipulating the activity of GPR35 could control the inflammatory response in colonic tissues (60).

GPR35 deficiency in intestinal epithelial cells leads to the reduction in both the number of goblet cells and the expression of Muc2, which occurs due to an increase in the pyroptosis of goblet cells (77). Pyroptosis is a form of cell death that contributes to inflammation, thus exacerbating inflammatory conditions. In consequence of this, the epithelial barrier is weakened, leading to increased susceptibility to infections such as those caused by Citrobacter rodentium (105, 106). In DSS-induced colitis mouse models, the specific deletion of GPR35 in macrophages resulted in elevated inflammation associated with a decrease in TNF production in macrophages (27). Although TNF is generally recognized for its pro-inflammatory role and is routinely targeted in treatments for IBD (107), it also serves an anti-inflammatory function. Specifically, it can induce the production of corticosterone in intestinal epithelial cells, a hormone that plays a key role in mitigating inflammation. The interplay between GPR35 and TNF provides new insights into the mechanisms through which inflammation can be regulated and controlled in gastrointestinal disorders (108).

Recently, it was discovered that when GPR35 was eliminated in the liver of non-alcoholic fatty liver disease (NAFLD) mice, there was an increase in inflammatory cytokines such as TNFα, IL6, and IL1β in the liver. This led to a worsening of hepatitis. However, the overexpression of GPR35 in the liver reversed these effects, hinting at its potential anti-inflammatory role in liver disease (109). Additionally, Kynurenic acid was found to improve energy metabolism and reduce inflammation in mice fed with a high-fat diet. However, TNF in adipose tissue increased in the absence of GPR35, and the anti-inflammatory effects of kynurenic acid were abolished via reducing anti-inflammatory cytokines such as IL-13, IL-4, IL-10, and IL-33 (74). This finding suggests that GPR35 might also exhibit anti-inflammatory effects in adipose tissue. Therefore, understanding the precise function of GPR35 could provide novel insights into inflammation in diseases like NAFLD and obesity. Interestingly, it is worth noting that kynurenic acid is recognized for its anti-inflammatory effects (74, 110, 111). However, kynurenic acid is known to interact with multiple receptors, not just GPR35, including α7 nicotinic acetylcholine receptor (a7nAChR), the aryl hydrocarbon receptor (AhR), and ionotropic glutamate receptors (112). Therefore, it is challenging to discern the specific influence of GPR35 in the observed anti-inflammatory effects of kynurenic acid.

In various tissues, different types of cells contribute to the anti-inflammatory effects orchestrated by GPR35. In the colorectum, it mainly operates through epithelial cells and goblet cells to exert anti-inflammatory effects. While it is known that GPR35 also facilitates anti-inflammatory in both the adipose tissue and the liver, the specific cell types involved in these processes have yet to be identified. When looking at immune cells, it was highlighted that the activation of GPR35 with kynurenic acid in macrophages leads to the suppression of NLRP3 inflammasome activation, consequently reducing related inflammation (49). Moreover, it was revealed that GPR35, along with its platelet- and mast-cell-derived ligand 5-HIAA, facilitates eosinophils recruitment to the lungs infected with cryptococcus neoformans and exacerbation of disease (82).

Overall, activation of GPR35 has been demonstrated to suppress the production of pro-inflammatory cytokines, stimulate the output of anti-inflammatory cytokines in a variety of tissues and organs, and fortify the integrity of the gut barrier. These findings highlight the multifaceted and context-dependent nature of GPR35 in inflammation. Nonetheless, a full understanding of the intricacies involved in GPR35 in counteracting anti-inflammatory demands further investigation.

## Conclusion and discussion

GPR35 is a distinctive G protein-coupled receptor with a significant role in the regulation of inflammation, demonstrating both pro-inflammatory and anti-inflammatory properties. Its pro-inflammatory effects have been witnessed in neutrophils, macrophages, and iNKT cells, while its anti-inflammatory attributes have been noted in varying contexts, including those in the colorectum, adipose tissue, and liver. Furthermore, GPR35 is increasingly recognized for its role in cancer and the related immune response. It facilitates angiogenesis within the tumor microenvironment, and GPR35 elimination in macrophages results in reduced immune cell infiltration in colon tumors (44). It was found that activating GPR35 in group 2 innate lymphoid cells fosters an immunosuppressive environment in lung cancer, thereby advancing the progression of lung cancer (113, 114). Consequently, GPR35 exhibits promising potential as a therapeutic target in cancer immunotherapy.

The question arises, “Could GPR35 be a potential target for treating inflammation-associated diseases?” The dual role of GPR35 in inflammation could have considerable consequences for devising therapeutic strategies centered on this receptor. For instance, the use of GPR35 agonists or antagonists could serve to regulate inflammatory responses in diverse diseases. In conditions characterized by excessive inflammation, such as IBD, inhibiting the pro-inflammatory actions of GPR35 could be advantageous. Conversely, in conditions requiring inflammation, such as wound healing or tissue repair, augmenting the anti-inflammatory effects of GPR35 activation may be beneficial. However, considering the intricate nature of GPR35 in inflammation, more comprehensive research is required to determine its exact impact and potential as a therapeutic target.

Currently, only two drugs targeting GPR35 have progressed to clinical trials. The first, Lodoxamide, has been employed for the treatment of allergic keratocon junctivitis (51), while the other, sodium cromoglycate (also known as RVT-1601 or PA101), is being used for treating idiopathic pulmonary fibrosis and chronic cough (115). Despite this, the potential for GPR35-focused treatment remains promising. GPR35 offers several advantages as a therapeutic target. Firstly, as a receptor situated on the cell surface, it is at the top of the signal transduction pathway. This is particularly significant given the numerous redundancies and compensatory mechanisms that exist within inflammation-related signal pathways, which can minimize these effects at the starting point.

Furthermore, GPR35 exhibits a tissue-specific expression pattern with high expression levels observed in immune cells and the gastrointestinal tract. The robust expression of GPR35 in immune cells aligns with its role in the regulation of inflammation. The substantial presence of GPR35 in the gastrointestinal tract, along with the strong correlation between GPR35 mutation and IBD, has led to the consideration of GPR35 as a potential target for IBD treatment. However, it is important to acknowledge the dual role GPR35 plays in IBD. While inhibiting GPR35 activity can diminish the proinflammatory response of macrophages and neutrophils, it may concurrently hinder the repair of gastrointestinal damage. Consequently, the risk-benefit balance of targeting GPR35 for IBD treatment remains somewhat elusive and requires further investigation.

The precise mechanisms driving the dual roles of GPR35 in promoting and inhibiting inflammation across various cells and tissues remain unclear and necessitate more in-depth research. The effects of GPR35 activation on inflammatory responses are likely shaped by numerous factors. These include cell types, tissue microenvironments, and the co-existence of other signaling pathways. Additionally, the expression levels and functional activity of GPR35 agonists may also be instrumental in steering the direction of the inflammatory response. In conclusion, GPR35 is a versatile receptor that can adopt either pro-inflammatory or anti-inflammatory stances within distinct immune cells and tissues. This warrants additional comprehensive research into GPR35 intricacies. Recently, the unveiling of the first protein structure of GPR35 marked a significant advancement in our understanding of GPR35 (86). As our knowledge of GPR35 deepens, we can anticipate the development and application of a broader range of GPR35-targeted drugs in the future.

## Author contributions

YW: Writing – original draft. PZ: Writing – original draft, Writing – review & editing. HF: Writing – review & editing. CZ: Writing – review & editing. PY: Writing – review & editing. XL: Conceptualization, Supervision. YC: Writing – review & editing, Conceptualization, Funding acquisition, Supervision.
